# Diet-induced changes in maternal gut microbiota and metabolomic profiles influence programming of offspring obesity risk in rats

**DOI:** 10.1038/srep20683

**Published:** 2016-02-12

**Authors:** Heather A. Paul, Marc R. Bomhof, Hans J. Vogel, Raylene A. Reimer

**Affiliations:** 1 Department of Biochemistry and Molecular Biology, University of Calgary, 3330 Hospital Drive NW, Calgary, AB, Canada, T2N 4N1; 2 Faculty of Kinesiology, 2500 University Drive NW, University of Calgary, Calgary, AB, Canada, T2N 1N4; 3 Department of Biological Sciences, Bio-NMR Center, 2500 University Drive NW, University of Calgary, Calgary, AB, Canada, T2N 1N4

## Abstract

Maternal obesity and overnutrition during pregnancy and lactation can program an increased risk of obesity in offspring. In this context, improving maternal metabolism may help reduce the intergenerational transmission of obesity. Here we show that, in Sprague-Dawley rats, selectively altering obese maternal gut microbial composition with prebiotic treatment reduces maternal energy intake, decreases gestational weight gain, and prevents increased adiposity in dams and their offspring. Maternal serum metabolomics analysis, along with satiety hormone and gut microbiota analysis, identified maternal metabolic signatures that could be implicated in programming offspring obesity risk and highlighted the potential influence of maternal gut microbiota on maternal and offspring metabolism. In particular, the metabolomic signature of insulin resistance in obese rats normalized when dams consumed the prebiotic. In summary, prebiotic intake during pregnancy and lactation improves maternal metabolism in diet-induced obese rats in a manner that attenuates the detrimental nutritional programming of offspring associated with maternal obesity. Overall, these findings contribute to our understanding of the maternal mechanisms influencing the developmental programming of offspring obesity and provide compelling pre-clinical evidence for a potential strategy to improve maternal and offspring metabolic outcomes in human pregnancy.

Obesity is now considered a global epidemic^1^. Importantly, maternal factors have been increasingly implicated in offspring susceptibility to obesity development, highlighting the impact of maternal adiposity and metabolism on the inter-generational transmission of obesity^2^. While improving maternal metabolism, in the case of maternal obesity, may help curb the burden of disease in future generations^3^, the maternal mechanisms through which beneficial or detrimental outcomes are programmed in offspring remain to be elucidated. It has also become clear that the gut microbiota greatly affects host metabolism^4^, and there is growing evidence that a dysbiotic microbiota plays a central role in the development of obesity^5^,^6^. Furthermore, maternal gut microbiota is an important factor in the colonization of the offspring gut^7^, impacting long-term metabolic health^8^. Elucidating the factors that influence the interplay of maternal obesity and the gut microbiota, and its impact on offspring obesity risk, may provide valuable insight into the mechanisms involved in the propagation of obesity across generations.

Dietary prebiotic treatment represents one strategy to selectively alter the substrates available to the gut microbiota and as a result affect host metabolism and physiology^9^. Prebiotics are nondigestible food ingredients that pass into the lower gastrointestinal tract where they stimulate the selective growth of health-promoting bacteria^10^,^11^. In the context of obesity, prebiotic intake is associated with reductions in body fat and energy intake^11^ and has been identified as a potential therapy for obesity management^12^. Furthermore, in diet-induced obese rats, prebiotics had a greater impact on body composition and gut microbial profiles than supplementation with a probiotic, a live microbial food ingredient^11^.

This study aimed to examine the metabolic and gut microbial effect of diet-induced maternal obesity in pregnant and lactating rats and the effect of supplementing a maternal high-fat/sucrose diet with the prebiotic oligofructose on maternal gut microbiota, metabolism, and offspring health. As a novel approach to maternal programming research, we employed ^1^H NMR serum metabolomics analysis to gain insight into the effect of diet-induced obesity and prebiotic supplementation on maternal metabolism; to assess the potential impact of the gut microbiota on the maternal metabolome; and to identify putative maternal metabolic signatures that might contribute to the developmental programming of offspring obesity.

Here we show that maternal prebiotic intake, in the context of diet-induced obesity, reduces gestational weight gain and prevents increased adiposity in both dams and offspring at weaning. These effects are accompanied by increased circulating concentrations of satiety hormones and abundance of *Bifidobacterium* spp. in the gut. Importantly, we demonstrate that diet-associated changes in maternal serum metabolite signatures provide novel insight into metabolic players involved in developmental programming of offspring obesity risk.

## Results

### Metabolic dysfunction following obesity induction

Female Sprague-Dawley rats underwent a 10 week obesity induction protocol using a high-fat/sucrose diet. By week 2 of obesity induction, high-fat/sucrose-fed females were significantly heavier than the age-matched lean reference group and remained so until mating (Supplementary Fig. S1). Diet-induced obese dams also had higher blood glucose and plasma insulin concentrations compared to lean dams during an oral glucose tolerance test (OGTT) that was performed following obesity induction (Supplementary Fig. S1). Obese dams displayed higher fasting plasma leptin, which is produced in proportion to fat mass, and lower peptide-YY (PYY), an appetite-reducing satiety hormone whose blood concentration is decreased in obesity in rats^13^ and humans^12^, compared to the lean reference group (leptin: obese 1162.7 ± 67.9 pmol L^−1^, lean: 749.5 ± 92.8 pmol L^−1^, P = 0.002; PYY: obese 13.7 ± 0.8 pmol L^−1^, lean 17.3 ± 1.1 pmol L^−1^, P = 0.028). Consistent with previous work^14^, diet-induced obesity altered gut microbiota composition, where obese dams had lower relative abundance of fecal *Bifidobacterium* spp. and higher relative abundance of *Clostridium* clusters XI and I as measured by real-time qPCR (Supplementary Table S1). Finally, whole-body metabolism of obese and lean dams was compared using ^1^H NMR serum metabolomics. Differences between metabolic profiles of obese and lean dams included variations in fatty acid oxidation (carnitine, o-acetylcarnitine, 2-hydroxybutyrate, ketone bodies), amino acid metabolism, gut microbial metabolites (propionate, formate, acetate, methanol), and phospholipid metabolism (cytidine) (Supplementary Fig. S2).

### Prebiotic normalizes gestational weight gain

**Figure 1:**
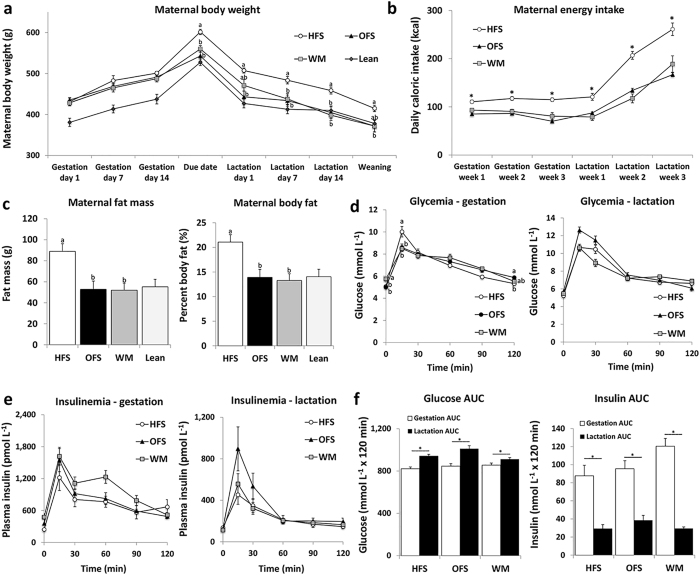
Effect of oligofructose supplementation on maternal body weight, energy intake, body composition, glycemia and insulinemia. During gestation and lactation, diet-induced obese dams were fed either a high-fat/sucrose diet (HFS obese control group), the high-fat/sucrose diet supplemented with 10% wt/wt oligofructose (OFS group), or a restricted amount of the high-fat/sucrose diet in order to match body weight to the OFS dams (WM). A lean reference group was maintained on control AIN-93 G diet through pregnancy and lactation for body weight and body composition measurements but was not included in statistical analysis. Maternal glycemia and insulinemia was determined from blood collected during oral glucose tolerance tests (OGTTs) performed on gestation day 14 and lactation day 19. Dams were fasted overnight (12 h) and an OGTT was performed after gavage with glucose (2 g/kg body weight). Blood samples were collected at 0, 15, 30, 60, 90 and 120 minutes during the OGTT. (**a**) Maternal body weight during gestation and lactation (HFS, n = 11; OFS, n = 12; WM, n = 9). (**b**) Maternal energy intake during gestation and lactation (HFS, n = 7; OFS, n = 12; WM, n = 9; Independent-Samples Kruskall-Wallis Test with adjusted significance where *P < 0.05 compared to all other groups). (**c**) Maternal fat mass and percent body fat at weaning (HFS, n = 10; OFS, n = 9; WM, n = 8). (**d,e**) Maternal glycemia and insulinemia (HFS, n = 7; OFS, n = 12; WM, n = 9). (**f**) Area under the curve (AUC) for glucose and insulin concentrations during the OGTTs (HFS, n = 7; OFS, n = 12; WM, n = 9; Student’s t-test). Graphs represent mean +/− SEM. Mean values without a common letter are significantly different using one-way ANOVA (P < 0.05). For body weight, glycemia, and insulinemia measurements, the data were analyzed using repeated measures one-way ANOVA, where the between subjects factor was maternal diet and the within-subjects factor was time. If a statistically significant interaction was observed, a one-way ANOVA between groups was performed.

To examine the effect of supplementing a maternal high-fat/sucrose diet with the prebiotic oligofructose, diet-induced obese dams were fed either a high-fat/sucrose diet *ad libitum* (obese control HFS group), the high-fat/sucrose diet supplemented with 10% wt/wt oligofructose *ad libitum* (OFS group), or a restricted amount of the high-fat/sucrose diet in order to match body weight to the OFS group (weight matched group, WM). The WM group was included so that the effect of oligofructose could be discerned independently from its known ability to lower body weight in the context of high-fat/sucrose diet-induced obesity^14^. Pre-pregnancy body weight did not differ between the maternally obese groups (HFS: 447.4 ± 7.2 g; OFS: 446.0 ± 7.2 g, WM: 439.3 ± 7.3 g, P = 0.738). Of the 42 diet-induced obese female rats, 32 mated successfully. Maternal body weight over gestation and lactation was dependent on maternal diet (time × diet interaction P = 0.001). HFS dams were heavier by their due date than both OFS and WM dams (Fig. 1a) and remained heavier than OFS dams until weaning, but were heavier than WM dams only on lactation days 7 and 14. As per experimental design, OFS and WM body weight did not differ throughout the study. To assess whether the disparity in maternal body weight between dietary groups was associated with differences in energy intake, food intake data was analyzed. Accordingly, HFS dams had the highest energy intake throughout pregnancy and lactation, while OFS and WM dams had similar energy intake (Fig. 1b). Body composition was measured using dual energy x-ray absorptiometry (DXA) at weaning, where HFS dams had higher fat mass and percent body fat compared to OFS and WM dams (Fig. 1c), indicating that the obese phenotype was maintained to a greater extent in HFS dams throughout pregnancy and lactation.

The effect of oligofructose on maternal glucose and insulin response was investigated on gestation day 14 and lactation day 19 via OGTTs. During gestation, WM dams had the highest fasting glucose, HFS dams had the highest peak glucose, and OFS dams had higher blood glucose than HFS dams at 120 minutes (Fig. 1d). There was no main effect of diet on maternal blood glucose during either gestation (P = 0.842) or lactation (P = 0.076). There were no differences in plasma insulin concentration during either gestation or lactation (Fig. 1e). However, maternal glucose area under the curve (AUC) increased in all groups from gestation to lactation, while insulin AUC decreased (Fig. 1f).

### Maternal prebiotic reduces offspring fat mass and glycemia

**Figure 2:**
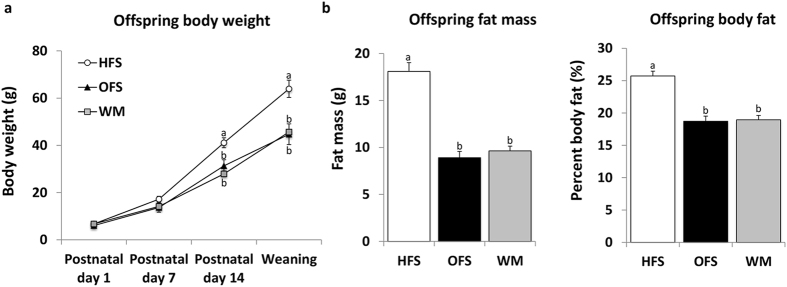
Effect of maternal oligofructose supplementation on offspring body weight and body composition. Pregnant and lactating diet-induced obese dams were fed either a high-fat/sucrose diet (HFS obese control group), the high-fat/sucrose diet supplemented with 10% wt/wt oligofructose (OFS group), or a restricted amount of the high-fat/sucrose diet in order to match body weight to the OFS dams (WM). Offspring body weight was measured weekly and body composition analyzed at weaning using DXA scan. Offspring body weight was calculated using litter averages as individual values. (**a**) Offspring body weight throughout lactation (HFS, n = 12; OFS, n = 12; WM, n = 9). (**b**) Offspring fat mass and percent body fat (HFS offspring, n = 31, 15 males and 16 females; OFS offspring, n = 29, 13 males and 16 females; WM offspring, n = 30, 13 males and 16 females). Values are mean ± SEM. Mean values without a common letter are significantly different using one-Way ANOVA (P < 0.05).

To determine the potential impact of a maternal high-fat/sucrose diet and oligofructose supplementation on offspring body weight and composition in early life, litters were weighed on postnatal days 1, 7, 14, and 21 (weaning), and body composition of, whenever possible, two male and two female offspring from each dam was measured via DXA scan at weaning. There was a significant interaction of offspring sex and body weight only at birth (P < 0.001), where male pups weighed more than female pups (males: 6.6 ± 0.1 g; females 6.2 ± 0.1 g, P = 0.001). HFS offspring weighed more than OFS and WM offspring by postnatal day 14 and remained heavier until weaning (Fig. 2a). HFS offspring had higher fat mass and percent body fat at weaning compared to OFS and WM offspring, which did not differ (Fig. 2b). These results were consistent with the increased plasma concentration of leptin in the HFS offspring (251.4 ± 17.8 pmol L^−1^) compared to both OFS offspring (113.3 ± 15.5 pmol L^−1^, P < 0.001) and WM offspring (133.3 ± 12.3 pmol L^−1^, P < 0.001). Fasting plasma glucose levels in HFS offspring (8.9 ± 0.3 mmol L^−1^) were significantly higher than both OFS (8.0 ± 0.2 mmol L^−1^, P = 0.013) and WM offspring (7.5 ± 0.1 mmol L^−1^, P < 0.001), which were not different. There were no differences in plasma insulin concentration among offspring (HFS offspring: 52.7 ± 5.3 pmol L^−1^; OFS offspring: 45.5 ± 4.6 pmol L^−1^; WM offspring: 58.8 ± 4.8 pmol L^−1^; P = 0.176). Finally, there was no difference in litter size between maternal groups throughout the study (HFS: 8.8 ± 0.4 pups; OFS: 8.8 ± 0.3 pups; WM: 8.6 ± 0.4 pups, P = 0.674), thus controlling for the potential influence of small versus large litter size on the programming of obesity in offspring^15^.

### Rapid changes in maternal gut microbiota

**Figure 3:**
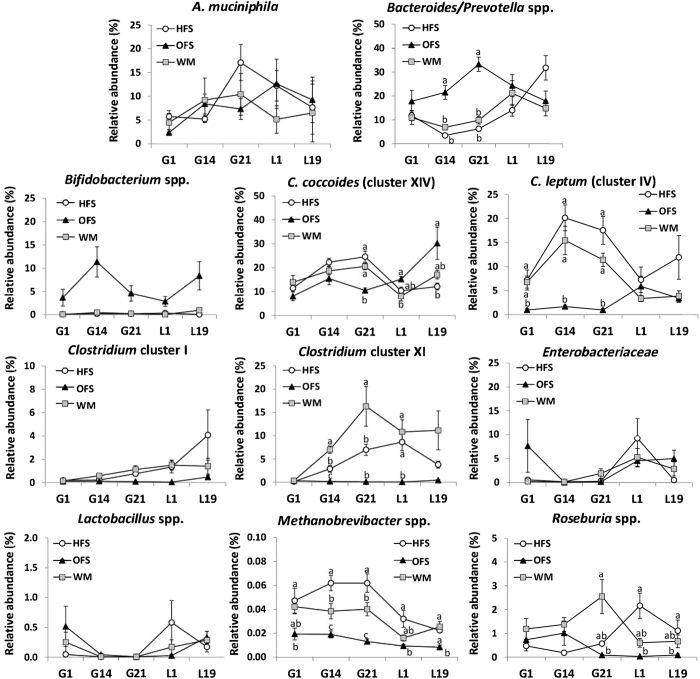
Relative microbial abundance of fecal microbiota changes throughout pregnancy and lactation and is affected by diet. Abundance of fecal microbiota from pregnant and lactating dams fed either a high-fat/sucrose diet (HFS obese control group), the high-fat/sucrose diet supplemented with 10% wt/wt oligofructose (OFS group), or a restricted amount of the high-fat/sucrose diet in order to match body weight to the OFS dams (WM) was determined using real-time qPCR. Microbial abundance was measured as 16 S rRNA gene copies per 20 ng DNA, and reported here as the relative abundance (%) of bacterial taxa per total bacteria. Longitudinal analysis of maternal fecal relative abundance on gestation days 1, 14, and 21 (G1, G14, G21), and lactation days 1 and 19 (L1, L19) was performed using repeated measures one-way ANOVA, where the between subjects factor was maternal diet and the within-subjects factor was timepoint (gestation days 1, 14 and 21 and lactation days 1 and 19). Values are mean ± SEM. Mean values without a common letter are significantly different using one-way ANOVA (P < 0.05) and indicate there was a significant interaction between time and maternal diet (P < 0.05). HFS, n = 11 except for *A. muciniphila* and *C. coccoides* where n = 10; OFS, n = 12; WM, n = 9.

To assess the impact of pregnancy and lactation and maternal diet on the composition of the maternal gut microbiota, we performed gut microbial profiling of maternal fecal samples collected on gestation days 1, 14 and 21, and lactation days 1 and 19. Relative abundance of *Bacteroides/Prevotella* spp., *Clostridium* cluster XI, *C. leptum*, *Enterobacteriaceae*, and *Methanobrevibacter* spp., changed significantly over gestation and lactation (main effect of time P < 0.05, Fig. 3), and approached significance in *Clostridium* cluster I (P = 0.052). Relative abundance of *Bacteroides/Prevotella* spp., *C. coccoides, C. leptum*, *Clostridium* cluster XI, *Methanobrevibacter* spp., and *Roseburia* spp. was dependent on maternal diet (time × diet interaction P < 0.05, Fig. 3). Across gestation and lactation, OFS dams had higher relative abundance of *Bifidobacterium* spp. and *Bacteroides/Prevotella* spp. (main effect of diet P < 0.05, Supplementary Table S2) compared to both HFS and WM dams. Notably, the increase in *Bifidobacterium* spp. in OFS dams was evident after 24 hours on the oligofructose supplemented diet (Fig. 3). Relative abundance of *Clostridium* cluster I differed only between HFS and OFS dams, while relative abundance of *Roseburia* spp. differed only between OFS and WM dams (Supplementary Table S2). The relative abundance of *C. leptum*, *Clostridium* cluster XI, and *Methanobrevibacter* spp. across gestation and lactation differed between all three maternal groups, although OFS dams had the lowest relative abundance. Finally, consistent with the fermentation of prebiotics by the gut microbiota, cecum weight was highest in OFS dams (OFS: 1.7 ± 0.2 g; HFS: 0.58 ± 0.04 g; WM: 0.67 ± 0.04 g; P < 0.001 versus both HFS and WM dams).

### Prebiotic offspring have distinct microbiota from HFS and WM

Maternal gut microbiota composition has been demonstrated to affect the colonization of offspring gut microbiota in early life, potentially affecting lifelong metabolic health^7^. Therefore, microbial profiling was also performed on cecal matter collected from offspring at weaning. Offspring sex affected the relative abundance of *Roseburia* spp. (P = 0.02) with males having higher relative abundance than females (males: 0.8 ± 0.3%; females: 0.15 ± 0.04%; P = 0.033). While maternal diet affected the relative abundance of 9 out of 11 microbial groups, in 4 out of 11 microbial groups (*Bifidobacterium* spp., *C. coccoides*, *C. leptum*, *Enterobacteriaceae*), HFS and WM offspring abundance were the same while OFS offspring differed (Table 1). WM offspring differed from both HFS and OFS offspring, which were the same, within *Clostridium* clusters I and XI, while HFS offspring differed from both OFS and WM offspring only within *Bacteroidetes/Prevotella* spp. As such, overall microbial profiles of OFS offspring were more distinct from HFS and WM offspring, which were more similar.

Finally, correlation analysis was used to assess the relationship between each measured maternal gut microbial group during the perinatal (gestation day 21 and lactation day 1) and weaning (lactation day 19) period with the corresponding offspring microbial abundance at weaning. Of the ten strongest correlations, seven were from the perinatal period (Table 2). Notably, the second strongest association was with *Bifidobacterium* spp. abundance from dams on lactation day 19. Overall, these data provide supporting evidence that maternal gut microbiota plays a role in the colonization of offspring gut microbial profiles in early life.

### Prebiotic increases maternal and offspring gut hormones

**Figure 4:**
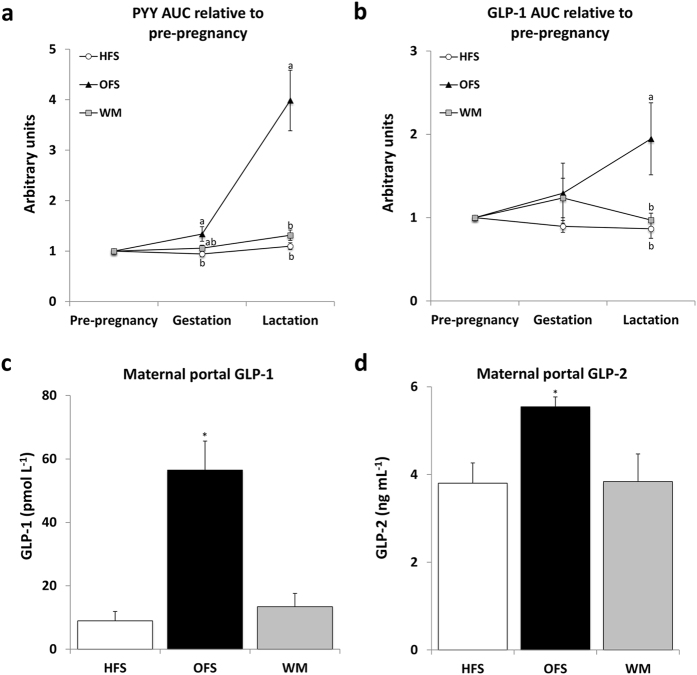
Oligofructose feeding increases circulating levels of peptide YY (PYY), glucagon-like peptide-1 (GLP-1) and glucagon-like peptide-2 (GLP-2). Blood samples for PYY and GLP-1 area under the curve were collected during the glucose tolerance tests performed pre-pregnancy, on gestation day 14, and lactation day 19 from diet-induced obese dams fed either a high-fat/sucrose diet (HFS obese control group), the high-fat/sucrose diet supplemented with 10% wt/wt oligofructose (OFS group), or a restricted amount of the high-fat/sucrose diet in order to match body weight to the OFS dams (WM) during pregnancy and lactation. Portal blood samples for GLP-1 and GLP-2 measurement were collected at euthanasia of the dams. (**a,b**) Relative increase of PYY and GLP-1 area under the curve (AUC) on gestation day 14 and lactation day 19 compared to pre-pregnancy AUC (PYY: HFS, n = 11; OFS, n = 12; WM, n = 9; GLP-1: HFS, n = 11; OFS, n = 10; WM, n = 9). (**c,d**) Portal plasma GLP-1 and GLP-2 concentration (GLP-1: HFS, n = 10; OFS, n = 9; WM, n = 7, GLP-2: HFS, n = 10; OFS, n = 9; WM, n = 8). Means without a common letter are significantly different using one-way ANOVA (P < 0.05). For relative AUC measurements, the data were analyzed using repeated measures one-way ANOVA, where the between subjects factor was maternal diet and the within-subjects factor was timepoint (pre-pregnancy, gestation and lactation). If a statistically significant interaction was observed, one-way ANOVAs between groups was performed. *P < 0.05 Independent-Samples Kruskall-Wallis Test with adjusted significance.

The reduction in energy intake in the OFS dams led us to examine whether circulating levels of the satiety hormones peptide-YY (PYY) and glucagon-like peptide-1 (GLP-1), both of which are associated with a reduction in food intake^16^,^17^ and increase upon prebiotic feeding^18^, were elevated in OFS dams. Relative to their pre-pregnancy levels, plasma PYY AUC increased in OFS dams to a greater extent compared to HFS dams by gestation day 14, and both WM and HFS dams by lactation day 19 (Fig. 4a). Plasma GLP-1 AUC also increased most in OFS dams (Fig. 4b). The gut trophic hormone glucagon-like peptide-2 (GLP-2) is co-secreted with GLP-1 by intestinal L-cells, is associated with intestinal health, and its secretion is also increased in response to prebiotic intake^19^. Accordingly, at weaning, portal plasma GLP-1 and GLP-2 were highest in OFS dams (Fig. 4c,d), confirming the influence of oligofructose fermentation on the secretion of glucagon-like peptides by the host. Finally, to test whether these satiety hormones were also increased in the offspring of OFS dams, we measured the concentration of PYY, GLP-1 and GLP-2 in cardiac plasma of offspring at weaning. Notably, plasma PYY, GLP-1, and GLP-2 were highest in OFS offspring compared to offspring of both HFS and WM dams (Table 3).

### Maternal body fat correlates with offspring body fat

Following gut microbial and satiety hormone profiles in offspring, we sought to determine what factors might be contributing to the reduced percent body fat in offspring of OFS and WM dams. While nine offspring variables were significantly correlated with offspring percent body fat, the variable that explained the largest amount of variance in offspring adiposity was maternal adiposity (R = 0.542, P < 0.01, Supplementary Table S3).

### Distinct maternal metabolomics signatures

Finally, we tested whether we could detect differences in whole-body metabolism between maternal groups during gestation and lactation, and thus identify potential maternal mechanisms involved in nutritional programming of offspring obesity risk. We therefore examined the differences in metabolite profiles between maternal groups using ^1^H NMR serum metabolomics analysis. Analysis was performed on serum collected on gestation day 14 and lactation day 19. In order to maximize differences between groups, multivariate analysis was performed using orthogonal partial least-squares discriminant analysis (OPLS-DA), where samples were clustered according to maternal diet. The explained variance (R^2^Y), predictability (Q^2^Y), and details of each model are described in Supplementary Table S4.

**Figure 5:**
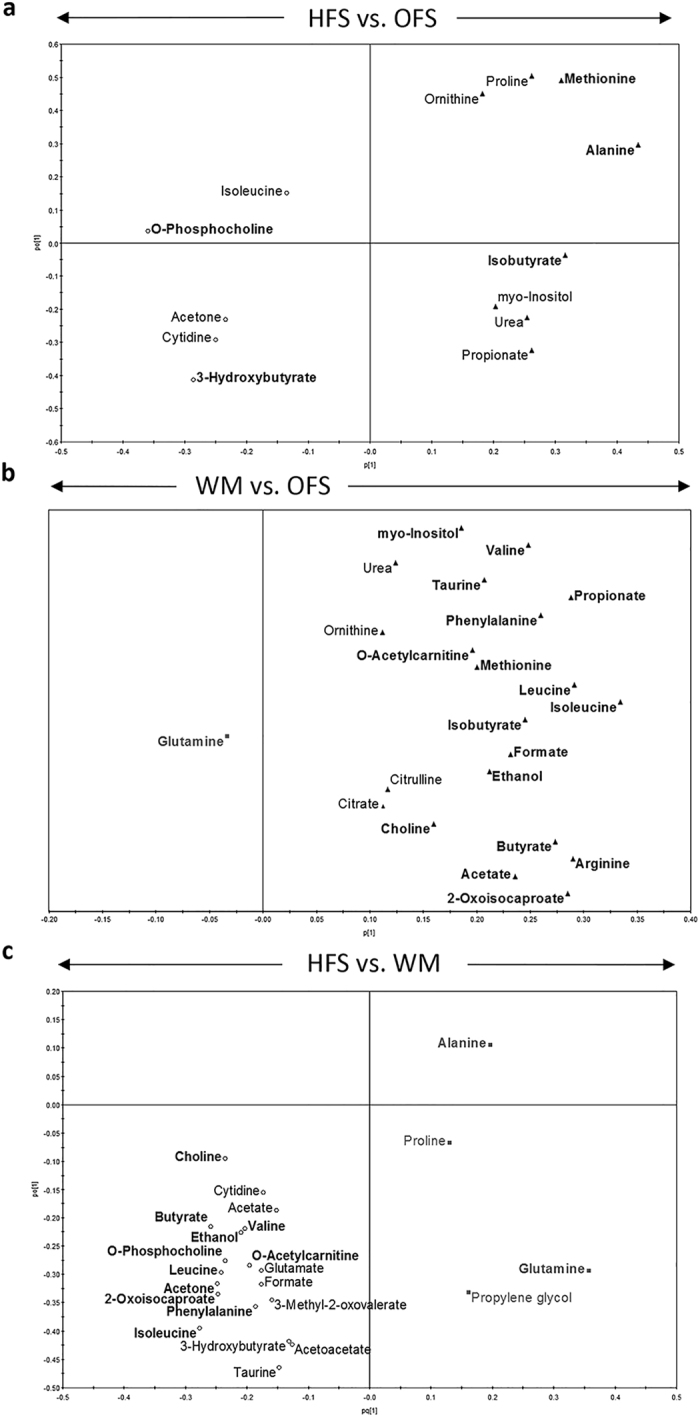
Metabolomic analysis of maternal serum collected during pregnancy. Comparison of maternal serum metabolites from dams fed a high-fat/sucrose diet (HFS obese control group), the high-fat/sucrose diet supplemented with 10% oligofructose (OFS group), or a restricted amount of the high-fat/sucrose diet to weight-match to the OFS group (WM) during gestation and lactation. (**a–c**) Pairwise OPLS-DA loadings plots comparing maternal serum metabolites from samples collected on gestation day 14. Metabolites included in the OPLS-DA modeling were selected following preliminary pairwise univariate analysis of the 57 metabolites identified and quantified by ^1^H NMR spectroscopy. Bold metabolites represent the variables that contributed most to the discrimination of dietary groups, which were identified using a combination of VIP > 1 and p(corr) > 0.4, with those contributing most to the separation located furthest from the center. The directions of the arrows indicate the maternal group corresponding to increased levels of those metabolites. HFS, n = 7; OFS, n = 12; WM n = 9.

Metabolomics analysis was able to clearly separate the maternal dietary groups during gestation (Fig. 5). Coinciding with the start point of the separation of body weight from HFS dams, metabolic profiles of OFS and WM dams were most similar, as indicated by the lowest R^2^Y and Q^2^Y (Supplementary Table S4). The strongest separation between maternal groups occurred between the HFS and the WM dams. HFS dams had increased levels of and precursors to ketone bodies (3-hydroxybutyrate, acetone, and acetoacetate, 2-oxoisocaproate) and metabolites involved in lipid metabolism (o-phosphocholine, cytidine). HFS dams also had elevated branched chain amino acids (isoleucine, leucine, valine) and decreased glucogenic amino acids (alanine, proline). OFS dams were characterized by altered levels of metabolites associated with changes in gut microbial composition, including short chain fatty acids (SCFAs) (isobutyrate, propionate, formate, butyrate, acetate, ethanol) and higher myo-inositol levels, a marker of increased insulin sensitivity^20^. WM dams displayed mainly increased glutamine and gluconeogenic substrates, including propylene glycol^21^. While there were general changes in amino acid metabolism across all maternal groups, OFS dams in particular exhibited increased levels of metabolites involved in arginine metabolism (arginine, ornithine, citrulline, proline). Interestingly, differences in choline and methionine occurred only during gestation, both of which are methyl donors and implicated in prenatal epigenetic histone methylation patterns in animal models^22^, potentially affecting the transgenerational transmission of obesity^23^.

**Figure 6:**
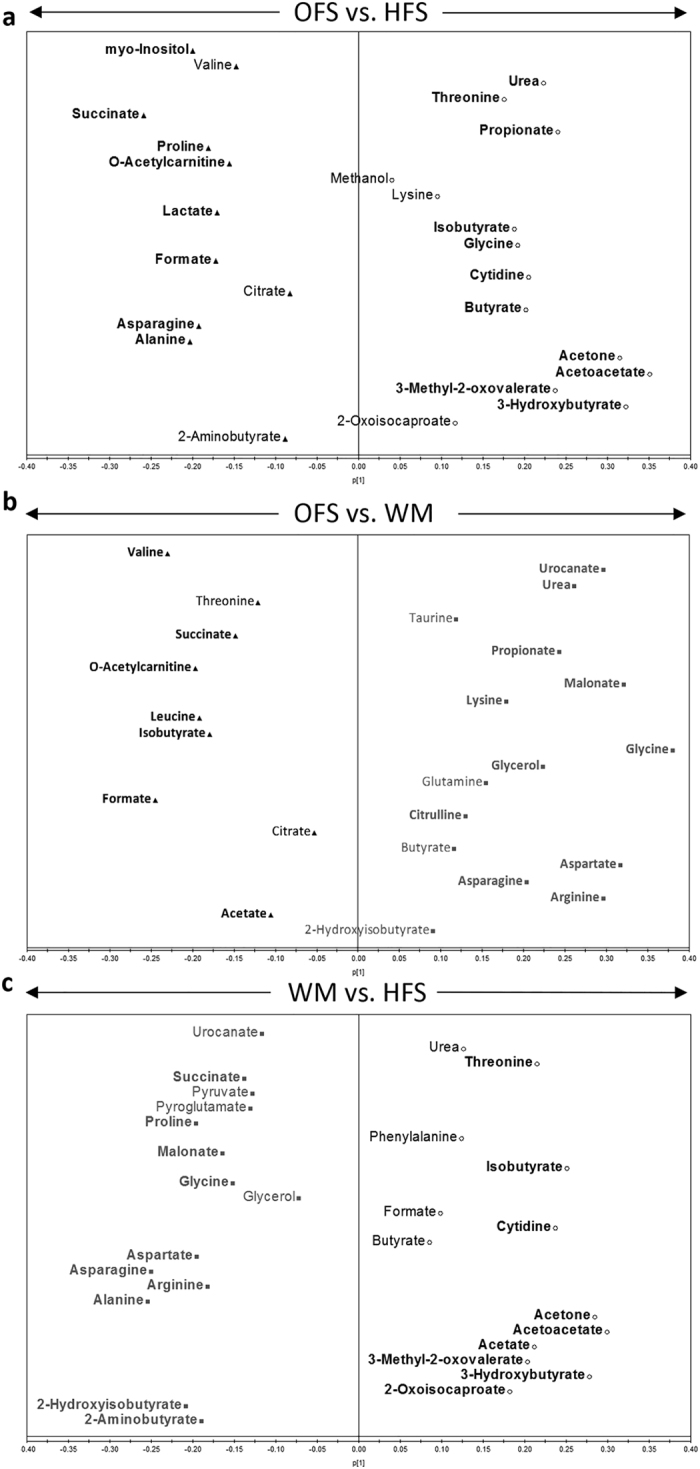
Metabolomic analysis of maternal serum collected during lactation. Comparison of maternal serum metabolites from dams fed a high-fat/sucrose diet (HFS obese control group), the high-fat/sucrose diet supplemented with 10% oligofructose (OFS group), or a restricted amount of the high-fat/sucrose diet to weight-match to the OFS group (WM) during gestation and lactation. (**a–c**) Pairwise OPLS-DA loadings plots comparing maternal serum metabolites from samples collected on lactation day 19. Metabolites included in the OPLS-DA modeling were selected following preliminary pairwise univariate analysis of the 57 metabolites identified and quantified by ^1^H NMR spectroscopy. Bold metabolites represent the variables that contributed most to the discrimination of dietary groups, which were identified using a combination of VIP > 1 and p(corr) > 0.4, with those contributing most to the separation located furthest from the center. The directions of the arrows indicate the maternal group corresponding to increased levels of those metabolites. HFS, n = 10; OFS, n = 9; WM, n = 8.

Figure 6 shows the metabolic profiles of lactating dams. Similar to gestation profiles, OFS and WM metabolic profiles in lactation were most similar, while HFS and WM dams were most distinct, indicated by their respective R^2^Y and Q^2^Y values (Supplementary Table S4). The modeled predictability (Q^2^Y) increased from gestation to lactation in all analyses, suggesting a strong effect of pregnancy itself on maternal metabolism.

In lactation, HFS dams continued to exhibit markers of increased ketone body synthesis, altered lipid metabolism, and a marker of impaired fasting glucose (3-methyl-2-oxovalerate)^24^. Finally, HFS dams had higher levels of threonine, an amino acid that may be elevated when the tricarboxylic acid (TCA) cycle slows in the presence of excess hepatic fatty acids^25^. OFS dams continued to be characterized by altered levels of SCFAs and increased myo-inositol, while increased levels of o-acetylcarnitine suggests decreased transport of fatty acids into the mitochondria. Finally, WM metabolic profiles were dominated by gluconeogenic substrates (glycine, alanine, aspartate, arginine, asparagine, proline, glycerol), markers of increased protein catabolism (urocanate), oxidative stress (2-aminobutyrate, pyroglutamate, taurine), *de novo* lipogenesis (malonate), and undernourishment (2-hydroxyisobutyrate).

Taken together, these data indicate that despite similar food intake, gestational weight gain, and percent body fat in OFS and WM dams, their serum metabolomics profiles diverged considerably and represented unique metabolite signatures. The reduced adiposity observed in offspring of both OFS and WM dams therefore is likely the consequence of distinct maternal metabolic processes, the long-term impact of which remains to be elucidated.

## Discussion

Early-life exposure of offspring to an adverse environment during critical periods in development, including the prenatal and suckling period, can increase the susceptibility to obesity later in life^2^. Improving maternal metabolism, however, may mitigate this malprogramming and thus decrease lifetime obesity risk. This study was designed to examine the potential benefit of supplementing diet-induced maternally obese dams with the prebiotic oligofructose and to determine whether this intervention could improve maternal metabolism in a manner that attenuates the detrimental metabolic programming of offspring due to maternal obesity. To our knowledge, this is the first study to examine the metabolomic impact of obesity in pregnant and lactating diet-induced obese Sprague-Dawley rats, especially in the context of prebiotic supplementation. Our main findings were that oligofructose supplementation decreased gestational weight gain and reduced both maternal and offspring adiposity, potentially due to the influence of maternal microbiota composition on maternal satiety and metabolism. These changes were associated with altered gut microbial and hormone profiles in offspring, potentially impacting their life-long obesity risk.

In this study, maternal adiposity at weaning showed a strong positive correlation with offspring adiposity. In humans, maternal weight gain during pregnancy is an independent predictor of infant adiposity^26^, where excess gestational weight gain increases life-long obesity risk in offspring^27^. As such, distinct patterns of weight gain between maternal groups during pregnancy likely contributed to the increased post-partum weight retention in HFS dams and the decreased percent body fat found in both OFS and WM dams and offspring at weaning.

Using ^1^H NMR metabolomics, we identified elevated circulating ketone bodies and branched-chain amino acids (BCAA) in the pregnant HFS dams. A key characteristic and driver of insulin resistance is increased basal lipolysis in adipose tissue, releasing free fatty acids (FFA) into circulation^28^. Hepatic β-oxidation then increases, where the resulting acetyl-CoA is metabolized to ketone bodies^29^. Accordingly, pregnant women exhibit increased circulating FFA and ketones; this is even more pronounced in women with gestational diabetes^30^. Higher BCAAs in pregnant women has also been associated with increased insulin resistance^31^. Our results therefore suggest increased insulin resistance in HFS compared to OFS and WM dams. This phenotype was also present during lactation, where HFS dams also displayed markers of impaired fasting glucose and altered TCA cycle metabolism. We posit that, during pregnancy, the circulating ketogenic environment in HFS dams contributed to the increased percent body fat of HFS offspring. The placenta is highly permeable to ketone bodies, which can subsequently be used as lipogenic substrates by offspring, increasing adipose tissue accretion^32^. In humans, maternal FFAs are also transported through the placenta and preferentially used for lipogenesis^32^. Furthermore, studies in both humans and rats have demonstrated that gestational diabetes is associated with increased maternal-fetal transfer of lipids across the placenta^32^, and that insulin resistance-associated disturbances in maternal lipid metabolism are independent factors in the developmental programming of compromised offspring metabolism^33^. Thus, elevated circulating FFA and resulting ketone bodies may play an important role in the early deposition of excess body fat in offspring.

Unexpectedly, gestational metabolic profiles of WM dams suggested both increased gluconeogenesis and mobilization of energy from muscle, rather than fat, a finding that is not consistent with healthy pregnancy^34^. This phenotype intensified during lactation, with the addition of markers of increased lipogenesis, stress, and undernourishment. This profile was in stark contrast to the OFS dams, despite similar daily energy intake. Rather, the metabolic profiles of the OFS dams were dominated by microbiota-associated SCFAs and, during lactation, markers of increased insulin sensitivity and decreased fatty acid import.

The SCFAs produced from the fermentation of oligofructose by gut bacteria ultimately enhances the secretion of the gut satiety hormones GLP-1 and PYY^18^. PYY binds to Y2 receptors on orexigenic NPY/AGRP neurons in the arcuate nucleus of the hypothalamus, inhibiting their effect, thus reducing hunger and food intake^16^. The incretin GLP-1 both suppresses appetite and improves glycemic response^17^. Here, PYY and GLP-1 levels are believed to have contributed to the reduced energy intake of OFS dams, thus reducing weight gain and improving insulin sensitivity. As a result, increased maternal satiety hormones in OFS dams may have played a role in reducing offspring adiposity. Notably, it may be the suppression of circulating satiety hormones PYY and GLP-1 in WM dams that played a role in the divergence of metabolic profiles observed in OFS and WM dams.

Diets high in sugar and fat, such as the high-fat/sucrose diet used in this study, activate food-reward and pleasure pathways in the brain, increasing motivation to eat^35^. Furthermore, caloric restriction results in a compensatory increase in appetite^36^, especially if preceded by obesity^37^. In contrast, bariatric surgery in humans increases GLP-1 and PYY, which is associated with decreased reward system activation and altered taste preference for palatable food^38^. Therefore, suppressed levels of GLP-1 and PYY in WM dams could perpetuate reward driven motivation for the high-fat/sucrose diet. However, due to their restricted food access, a perceived lack of adequate energy intake to meet the metabolic demands of pregnancy and lactation could trigger a shift in their metabolism to reprioritize energy provision away from developing and suckling pups to the dam. Thus, the metabolic response identified in the WM dams might represent a stress-induced state, although this remains speculative. Finally, it has also been shown that caloric restriction in lactating dams lowers milk supply, resulting in reduced offspring body weight^39^. Altogether, these data suggest that the reduced adiposity observed in WM offspring may have been due to the favoring of maternal needs over those of the pups^40^ and decreased milk availability.

Arginine, ornithine, citrulline, and proline belong to interrelated metabolic pathways involving arginine metabolism, which is crucial to proper placental and fetal growth in rats^41^; elevated circulating levels of arginine and its metabolites have been implicated in improved pregnancy outcomes in mammals and reduced adiposity in offspring^42^. It is possible that differences in maternal arginine metabolism played a role in decreasing adiposity in OFS offspring, especially as both overfed and underfed dams have demonstrated impaired placental transport of arginine and ornithine, compromising placental function^43^. While it is difficult to determine the reason for increased arginine in OFS dams during pregnancy, improved maternal gut health in OFS dams may have resulted in increased citrulline synthesis by intestinal epithelial cells, a potential marker of gut health, which can then be converted to arginine via renal metabolism^44^. This effect would be consistent with the increased secretion of the gut trophic hormone GLP-2 by intestinal cells, which is co-secreted with GLP-1^19^ and was increased in the portal plasma of OFS dams at weaning.

Recently, Koren *et al.*^45^ demonstrated that the gut bacterial composition of pregnant women changes radically from the first to the third trimester, an effect driven by host-microbial interactions. These changes are purported to aid in the metabolic adaptations in insulin sensitivity and adiposity required for a healthy pregnancy. Similarly, we found that the abundance of maternal gut microbiota changed throughout pregnancy. Further, oligofructose supplementation induced shifts in the overall pattern of gut microbiota during pregnancy compared to HFS and WM dams, notably within the *Bifidobacterium* and *Clostridium* spp., a finding consistent with oligofructose intake in rats^11^. While the specific metabolic effects of other microbial groups investigated in this study remain unclear, the health benefits of bifidobacteria are well established^17^ as is their predominance in the infant gut^46^,^47^. Further, studies have indicated that there is intestinal transfer of bifidobacteria^47^,^48^ and other microbes^45^,^49^ from mother to infant during vaginal birth^50^. In the context of maternal diet, a recent metagenomic study in primates reported that a maternal high-fat diet induced persistent changes in offspring gut microbial composition, and that these changes likely influenced offspring metabolism up to one year of age^51^. Our correlation data supports this potential impact of maternal gut microbiota during the perinatal period on the colonization of the offspring gut. Further, studies in mice have linked maternal prebiotic intake with alterations in offspring microbiota^52^. Therefore, modifying the high-fat/sucrose-induced maternal gut microbiota composition with oligofructose may have helped establish a more healthful profile in OFS offspring. Here, offspring sex affected the relative abundance of *Roseburia* spp. These results may have future relevance for the offspring as recent research suggests that sex-specific differences in the gut microbiome influence immune function and disease susceptibility^53^. Whether the differences observed in this study at weaning might affect long-term sex-specific susceptibility to obesity remains to be determined. Finally, there is a lack of research regarding the stability of the maternal gut microbiota during lactation. It is possible, though, that the changes in microbial abundance throughout lactation seen in this study were associated with the corresponding decrease in body weight and adiposity that occurred.

Evidence that the metabolic benefits in the OFS dams and their offspring were mediated via the gut microbiota is three-fold. First, OFS dams and their offspring showed marked differences in their gut microbial composition. Consistent with the bifidogenic effect of oligofructose^17^, bifidobacteria abundance was high only in the OFS dams and their offspring. Second, shifts in gut microbiota composition and activity affect the host serum metabolome^4^,^9^, including circulating levels of SCFAs^54^, the metabolic end-products of oligofructose fermentation. As the metabolic profiles of OFS dams were dominated by shifts in multiple SCFAs, it is plausible that the OFS serum metabolome was greatly influenced by the gut microbiota. Notably, a recent human study found that circulating levels of maternal SCFAs during late pregnancy were associated with differences in maternal weight gain and newborn body weight^54^. Finally, SCFAs are believed to mediate the enhanced secretion of the satiety hormones PYY and GLP-1^11^. Circulating levels of these satiety hormones not only increased in OFS dams as pregnancy and lactation progressed, but were also increased in their offspring.

Both oligofructose supplementation and the weight-matching protocol profoundly influenced the metabolic profiles of maternally obese dams with ^1^H NMR serum metabolic profiling playing a central role in defining the metabolic advantages of OFS supplementation compared to both *ad-libitum* and restricted high-fat/sucrose feeding. Likewise, metabolomics analysis identified a number of metabolite signatures potentially implicated in programming of offspring metabolism and demonstrates the potential of metabolomics to increase understanding of the molecular mechanisms involved in developmental programming of health and disease. Metabolic profiling also highlighted the potential for maternal gut microbiota to influence circulating metabolites. As such, it provides evidence that the influence of the maternal gut microbiota on offspring health is not limited to intestinal colonization with microbiota, but also includes its impact on overall maternal metabolism, an effect that ultimately may alter offspring metabolic risk.

In conclusion, our data suggests that supplementing pregnant and lactating obese rats with oligofructose improved maternal metabolism in a manner that ultimately decreased adiposity in offspring. These benefits were likely mediated via prebiotic-induced changes in maternal gut microbiota composition. Whether these findings translate to the human condition, for example whether prebiotics may help women with obesity achieve healthy weight gain during pregnancy and reduce postpartum weight retention, remains to be determined. It is clear, however, that there exists a strong interplay between maternal satiety, energy intake, gut hormones, gut microbiota, and circulating metabolites, which ultimately influences the developmental programming of offspring obesity risk. Further examination of these mechanisms, especially in humans, is warranted and may inform strategies to decrease the propagation of obesity across generations.

## Methods

### Animals and diets

The study protocol was approved by The University of Calgary Animal Care Committee and conformed to the *Guide for the Care and Use of Laboratory Animals.* One hundred and four 12-week-old virgin female Sprague Dawley rats were obtained from Charles River (Charles River, St. Constant, QC) and housed on a 12 h light-dark cycle in a controlled environment. Rats (n = 90) were fed a high-fat/sucrose diet (Diet #102412, Dyets, Inc) *ad libitum* for 10 weeks to induce obesity. The 42 rats with the greatest weight gain were allocated to one of three groups throughout gestation and lactation in a manner that ensured that the pre-pregnancy body weight of each maternal group was not significantly different (n = 14 per group): 1) High-fat/sucrose *ad libitum* (obese control HFS group); 2) high-fat/sucrose + 10% wt/wt oligofructose (Orafti P95, BENEO-Orafti Inc.) *ad libitum* (OFS group); 3) weight matched to the OFS group with limited high-fat/sucrose diet provision (WM). The amount of high-fat/sucrose diet provided to the WM dams was based on a combination of repeated body weight measurements of both the WM and OFS dams and caloric intake measurements from the OFS dams. A fourth lean reference group (n = 14) was maintained on control AIN-93 diet throughout the study. Group size was selected using a power calculation based on expected differences between groups for oligofructose-induced changes in gut hormones and anticipated maternal fecundity and pregnancy outcomes. Diet composition is described in Supplementary Table S5. Following obesity induction, females were bred with male Sprague-Dawley rats in wire-bottomed cages. The day a copulation plug was found was designated as gestation day 0, at which point the females were housed individually and provided their experimental diets. Within 24 hours of parturition, litters were culled to 10 pups with equal numbers of males and females; extra pups were cross-fostered to dams with less than 10 similar-aged pups when possible. Maternal and pup body weight was measured weekly and maternal food intake measured daily throughout pregnancy and lactation. At weaning, dams and, when possible, two male and two female pups per dam were lightly anaesthetized with isoflurane and body composition measured via dual energy x-ray absorptiometry (DXA) scan with software for small animals (Hologic ODR 4500; Hologic). Final tissue collection in the pups and dams occurred following the DXA scan. Animals were euthanized with anesthetic overdose and aortic cut. Maternal fecal samples were collected pre-pregnancy, on gestation days 1, 14, and 21, and on lactation days 1 and 19.

### Oral glucose tolerance tests/metabolomics sample collection

Maternal glycemia and insulinemia was assessed using oral glucose tolerance tests (OGTTs) as per our previous work^11^ on gestation day 14 and lactation day 19. Prior to the OGTTs, following the overnight fast, blood was collected via saphenous vein puncture and the serum stored at -80°C for later metabolomics analysis.

### Final plasma and tissue collection

Following an overnight fast, rats were anaesthetized with isoflurane and portal blood (dams) or cardiac blood (offspring) samples were collected into chilled tubes containing inhibitors as per our previous work^11^. Rats were euthanized by overanesthetization and aortic cut. Dam liver and cecum were excised and weighed. Cecal matter from offspring was collected. All samples were stored at -80°C until analysis.

### Plasma satiety hormones

Portal plasma GLP-1 and GLP-2 was measured using ELISA (Millipore, Billerica, MA). Insulin, leptin, ghrelin (active), GIP (total), PYY, and GLP-1 (active) were quantified using the Rat Gut Hormone Multiplex Kit (Millipore) from blood collected during the OGTTs. Offspring plasma glucose concentrations were determined using the Glucose Liquicolor assay (Stanbio Laboratory, Boerne, TX).

### Gut microbiota profiling

Microbial profiling was performed using real-time qPCR according to our previous work^55^.

### Preparation, analysis and profiling of maternal serum samples for ^1^H NMR

Samples were prepared, analyzed, and profiled in a randomized order. Serum samples were thawed and 200 μL filtered using pre-washed 3-kDa NanoSep microcentrifuge filters. The filtrate was transferred to a clean microcentrifuge tube, 10uL of sodium azide (NaN_3_) and 80 μL of phosphate buffer in D_2_O containing dimethyl silapentane sulfonate (DSS, final concentration 0.5 mmol L^−1^) were added, samples were adjusted to a pH of 7.0 ± 0.02, and then brought to a final volume of 400 μL with D_2_O. Spectra were acquired using a standard pulse program (prnoesy1d) on a Bruker Advance 600 spectrometer (Bruker Biospin, Milton, Canada) operating at 600.22 MHz at 297 K (5 mm TXI Probe)^56^, individually processed using Chenomx NMR Suite 7.5 software (Chenomx Inc., Edmonton, Canada), and targeted profiling completed using the Chenomx NMR Suite profiling module^57^.

### Metabolomics analysis

A total of 57 metabolites were quantified in each sample, followed by normalization to the total sum of metabolite concentrations. Normalized values were subjected to preliminary pairwise univariate analysis and metabolites with a P < 0.3 were selected for multivariate analysis using SIMCA-P + 12.0.1 software (Umetrics)^58^. Principal component analysis was used to detect and remove outliers. To cluster observations within dietary groups, pair-wise orthogonal partial least-squares discriminant analysis (OPLS-DA) was performed, with k-fold cross-validation where k ranged from 5 to 7 depending on the number of samples(n) so that n/k ≥ 3 (Supplementary Table S4). All models were generated using unit variance scaling. OPLS-DA analysis was performed using the normalized metabolite concentrations as the X matrix and the maternal dietary group as the Y predictors. OPLS-DA model fit and predictability was assessed using R^2^Y and Q^2^Y values, where an R^2^Y value of 1 indicates that the model explains 100% of the variation in metabolites between groups, and a Q^2^Y of 1 indicates total reliability of the prediction during cross-validation^58^. All models were checked for over-fitting by ensuring that the difference between R^2^Y and Q^2^Y did not exceed 0.3^58^. The metabolites that contributed most to the characterization of the dietary groups were chosen based on a combination of a variable importance to projection (VIP) value cut-off > 1 and a p(corr) > 0.4^59^.

### Statistical Analyses

Results are reported as mean ± SEM. Maternal cross-sectional data was tested for normality using the Shapiro-Wilk test. If normally distributed, differences between groups were evaluated using either Student’s t-test or one-way analysis of variance (ANOVA) with Tukey’s *post-hoc* tests as appropriate. If normality was violated, data was subjected to non-parametric pairwise analysis with adjusted significance. Equality of variance was tested using Levene’s test. If homogeneity of variance was violated, the corresponding modified P value was used. Longitudinal and OGTT-derived data was assessed using repeated measures ANOVA. For offspring, the effect of sex and maternal diet was evaluated using a two-way ANOVA. If there was no significant effect of sex, data for males and females were pooled. All cross-sectional offspring outcomes were analyzed using one-way ANOVA. For correlation analysis, Pearson’s correlation was used when comparing normally distributed data; otherwise Spearman’s correlation was used. Student’s t-tests or one-way ANOVAs were used to assess the main effect of diet in all gut microbial analysis. In all tests, P < 0.05 was considered significant. Statistical analyses were performed using SPSS version 20.0.0 software (SPSS, Inc., Chicago, IL, USA). The lean reference group was not included in statistical analyses after the obesity induction period.

## Additional Information

**How to cite this article**: Paul, H. A. *et al.* Diet-induced changes in maternal gut microbiota and metabolomic profiles influence programming of offspring obesity risk in rats. *Sci. Rep.* **6**, 20683; doi: 10.1038/srep20683 (2016).

## Supplementary Material

Supplementary Information

## Figures and Tables

**Table 1:** Relative abundance of cecal gut microbiota from offspring of maternally obese dams fed different diets during pregnancy and lactation.

Microbial Group	Offspring Relative Microbial Abundance (%)		
	HFS	OFS	WM
*Akkermansia muciniphila*	1.1 ± 0.3^a^	0.5 ± 0.2^ab^	0.22 ± 0.05^b^
*Bacteroides/Prevotella* spp.	27.6 ± 2.0^b^	36.9 ± 1.6^a^	34.2 ± 1.8^a^
*Bifidobacterium* spp.	0.010 ± 0.001^b^	0.4 ± 0.1^a^	0.015 ± 0.001^b^
*Clostridium coccoides* (cluster XIV)	4.9 ± 0.3^b^	8.5 ± 0.6^a^	6.0 ± 0.4^b^
*Clostridium leptum* (cluster IV)	1.3 ± 0.2^a^	0.24 ± 0.03^b^	1.4 ± 0.2^a^
*Clostridium* cluster I	0.14 ± 0.04^b^	0.08 ± 0.02^b^	0.45 ± 0.06^a^
*Clostridium* cluster XI	0.06 ± 0.02^b^	0.05 ± 0.01^b^	0.26 ± 0.04^a^
*Enterobactericeae*	0.6 ± 0.1^b^	1.6 ± 0.3^a^	0.47 ± 0.06^b^
*Lactobacillus* spp.	0.19 ± 0.04	0.3 2± 0.08	0.22 ± 0.02
*Methanobrevibacter* spp.	0.012 ± 0.001^c^	0.025 ± 0.002^b^	0.03 ± 0.002^a^
*Roseburia* spp.	0.5 ± 0.2	0.05 ± 0.02	0.8 ± 0.3

Values are mean ± SEM expressed as the relative abundance (%) of bacterial taxa per total bacteria. Mean values without a common letter are significantly different (P < 0.05) and denote a main effect of maternal diet when analyzed by one-way ANOVA. HFS offspring, n = 29, 15 males and 14 females; OFS offspring, n = 29, 13 males and 16 females; WM offspring, n = 29, 14 males and 15 females.

**Table 2:** Top ten correlations between microbial abundance of offspring cecal microbiota at weaning and maternal fecal microbiota during the perinatal and weaning period.

Microbial Group	Maternal Fecal Sample	R*
*C. leptum*	Gestation day 21	0.694
*Bifidobacterium* spp.	Lactation day 19	0.628
*Bacteroidetes/Prevotella* spp.	Gestation day 21	0.498
*Roseburia* spp.	Gestation day 21	0.441
*Methanobrevibacter* spp.	Gestation day 21	−0.431†
*Bifidobacterium* spp.	Gestation day 21	0.408
*C. coccoides* (cluster XIV)	Lactation day 19	0.381
Total Bacteria	Gestation day 21	0.364†
*C. coccoides* (cluster XIV)	Lactation day 1	0.355
Total Bacteria	Lactation day 19	0.343

^*^All correlations P < 0.01.

^†^ = Pearson’s correlation value.

**Table 3:** Offspring gut and satiety hormone levels at weaning.

Maternal Diet	PYY (pmol L^−1^)	GLP-1 (pmol L^−1^)	GLP-2 (ng mL^−1^)
HFS	20.3 ± 1.2^b^	32.4 ± 5.2^b^	0.9 ± 0.1^b^
OFS	24.6 ± 1.5^a^	71.8 ± 5.6^a^	1.6 ± 0.1^a^
WM	19.4 ± 1.1^b^	51.2 ± 6.9^b^	1.1 ± 0.1^b^

Values are mean ± SEM. Mean values without a common letter are significantly different (P<0.05) using one-way ANOVA. HFS: n=31, 16 males and 15 females, OFS: n=29, 13 males and 16 females; WM: n=30, 15 males and 15 females.
