# Supplementary material for: Diet-induced changes in maternal gut microbiota and metabolomic profiles influence programming of offspring obesity risk in rats
Source: Sci Rep. 2016 Feb 12;6:20683. doi: 10.1038/srep20683 (PMC4751613; doi:10.1038/srep20683)
Supplement: Supplementary Information [file srep20683-s1.pdf]

**Supplementary Information for Manuscript**: Diet-induced changes in maternal gut microbiota and metabolomic profiles influence programming of offspring obesity risk in rats

**Authors:** Heather A. Paul, Marc R. Bomhof, Hans J. Vogel, Raylene A. Reimer*

*corresponding author; E-mail: reimer@ucalgary.ca; Tel: 1 (403) 220-8218

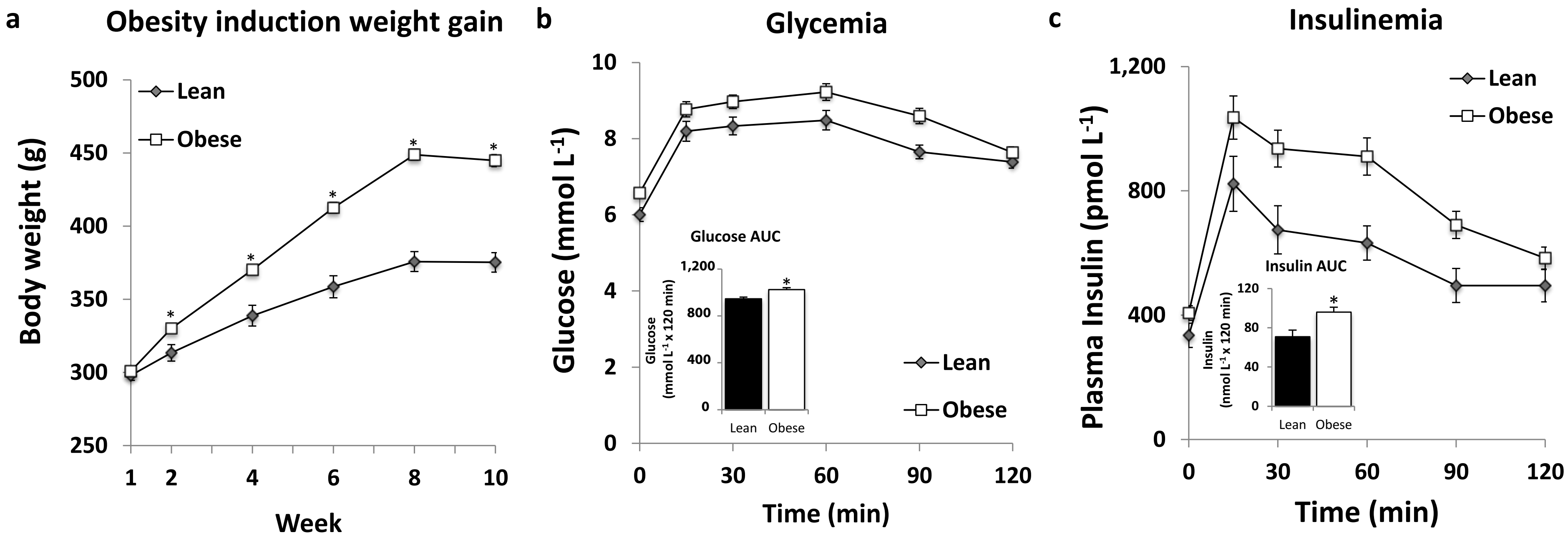


**Supplementary Figure S1**. A high-fat/sucrose diet increases body weight, glycemia and insulinemia in female Sprague-Dawley rats. Obesity was induced in 12-week old female Sprague-Dawley rats for 10 weeks using a high-fat/sucrose diet. Dams were fasted overnight (12 h) and an OGTT was performed after gavage with glucose (2g/kg body weight). Blood samples were collected at 0, 15, 30, 60, 90 and 120 minutes during the OGTT for glucose levels and insulin determination. (a) High-fat/sucrose feeding increased body weight by week 2 (b,c) Glucose and insulin levels and area under the curve (AUC) during the OGTT. Data were analyzed using repeated measures one-way ANOVA, where the between subjects factor was diet and the within-subjects factor was time. If a statistically significant interaction was observed, Student's t-tests between groups was performed. Lean: *n*=14, Obese: *n*=42.
* $P<0.05$ compared to lean dams.

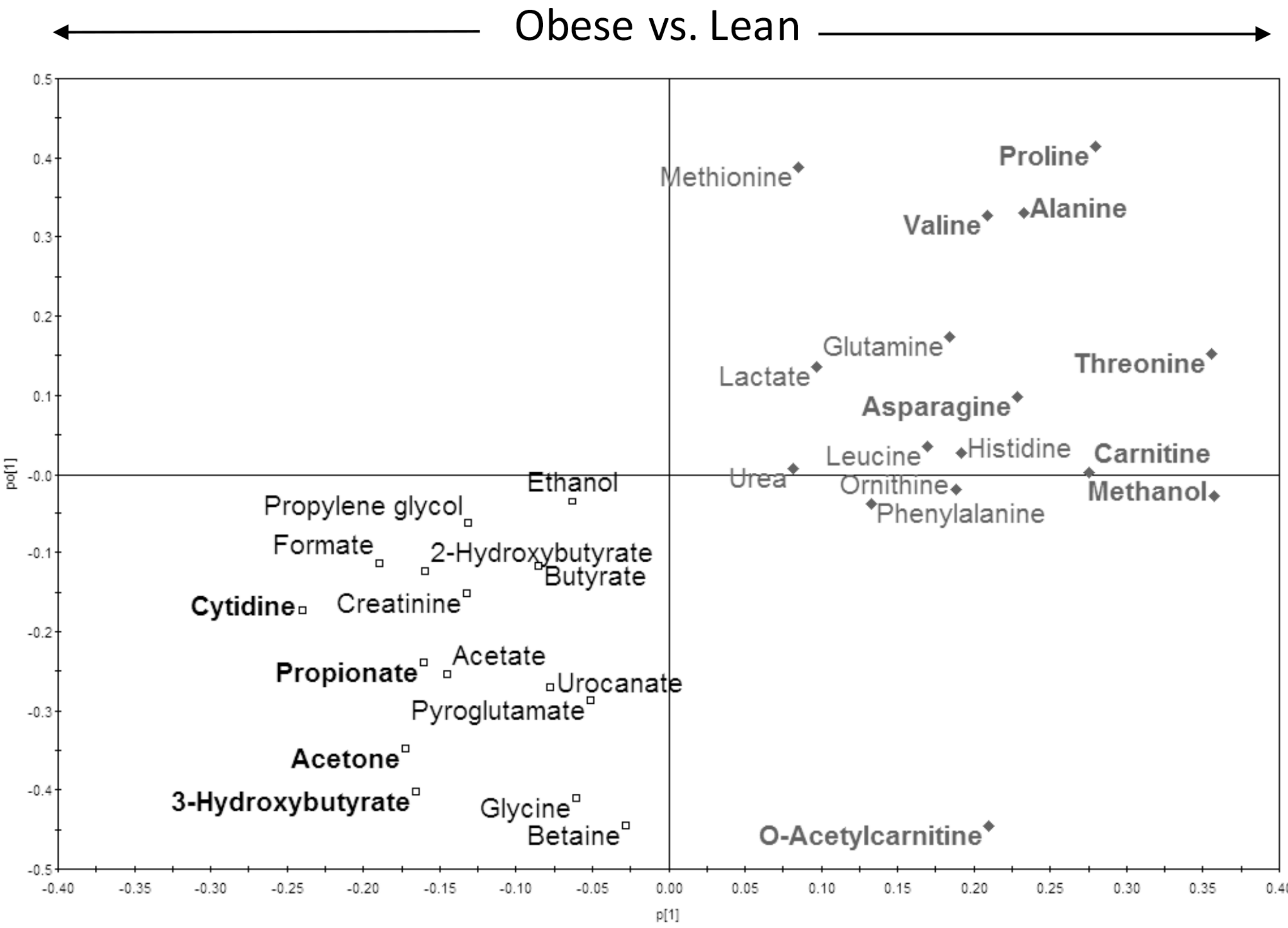


**Supplementary Figure S2**. OPLS-DA loadings plot comparing serum metabolites from dams fed a high-fat/sucrose diet or a lean AIN-93M diet for 10 weeks. Metabolites included in the OPLS-DA model were selected following preliminary pairwise univariate analysis of the 57 metabolites identified and quantified by $^{1}$H NMR spectroscopy. Bold metabolites represent the variables that contributed most to the discrimination of dietary groups, which were identified using a combination of VIP>1 and p(corr) >0.4, with those contributing most to the separation located furthest from the center. The directions of the arrows indicate the maternal group corresponding to increased levels of those metabolites. Lean: *n*=14; Obese: *n*=39.

**Supplementary Table S1**. Relative abundance of fecal gut microbiota following a 10 week obesity induction period in female Sprague-Dawley rats.

| | **Relative Microbial Abundance (%)** | |
|---|---|---|
| **Microbial Group** | **Lean** | **Obese** |
| *Akkermansia muciniphila* | 6.7 ± 1.4 | 9.7 ± 1.2 |
| *Bacteroides/Prevotella* spp. | 2.4 ± 0.4 | 3.5 ± 0.4 |
| *Bifidobacterium* spp. | 1.4 ± 0.4* | 0.033 ± 0.006 |
| *Clostridium coccoides* (cluster XIV) | 28.6 ± 2.1 | 26.9 ± 1.3 |
| *Clostridium leptum* (cluster IV) | 25.5 ± 2.6 | 20.8 ± 1.8 |
| *Clostridium* cluster I | 0.36 ± 0.04 | 1.3 ± 0.2* |
| *Clostridium* cluster XI | 2.3 ± 1.3 | 7.7 ± 1.4* |
| *Enterobacteriaceae* | 0.8 ± 0.3 | 0.5 ± 0.1 |
| *Lactobacillus* spp. | 0.5 ± 0.2 | 0.013 ± 0.003 |
| *Methanobrevibacter* spp. | 0.034 ± 0.002 | 0.038 ± 0.002 |
| *Roseburia* spp. | 0.07 ± 0.02 | 0.21 ± 0.05 |

Values are mean ± SEM expressed as the relative abundance (%) of bacterial taxa per total bacteria (16S rRNA gene copies of microbial group/total bacteria 16S rRNA gene copies); *n*=14 (lean), *n*= 42 (obese). *Significantly different between lean and obese, Student's t-test ($P<0.05$).

**Supplementary Table S2**. Average relative abundance of maternal fecal microbiota across gestation and lactation.

| | Relative Maternal Microbial Abundance (%) | | |
|---|---|---|---|
| **Microbial Group** | **HFS** | **OFS** | **WM** |
| *Akkermansia muciniphila* | 9.6 ± 1.6 | 8.0 ±1.6 | 7.1 ± 1.8 |
| *Bacteroides/Prevotella* spp. | 13.5 ± 1.8[b] | 22.9 ± 1.8[a] | 12.7 ± 1.5[b] |
| *Bifidobacterium* spp. | 0.20 ± 0.03[b] | 6.2 ± 1.1[a] | 0.37 ± 0.09[b] |
| *Clostridium coccoides* (cluster XIV) | 16.1 ± 1.1 | 15.9 ± 1.8 | 15.6 ± 1.1 |
| *Clostridium leptum* (cluster IV) | 12.8 ± 1.5[a] | 2.6 ± 0.5[c] | 8.2 ± 1.0[b] |
| *Clostridium* cluster I | 1.3 ± 0.5[a] | 0.16 ± 0.04[b] | 0.9 ± 0.2[ab] |
| *Clostridium* cluster XI | 4.5 ± 0.6[b] | 0.23 ± 0.04[c] | 9.1 ± 1.5[a] |
| *Enterobacteriaceae* | 2.1 ± 0.9 | 3.5 ± 1.2 | 2.1 ± 0.6 |
| *Lactobacillus* spp. | 0.16 ± 0.08 | 0.18 ± 0.07 | 0.14 ± 0.05 |
| *Methanobrevibacter* spp. | 0.045 ± 0.004[a] | 0.014 ± 0.001[c] | 0.032 ± 0.003[b] |
| *Roseburia* spp. | 0.9 ± 0.2[ab] | 0.4 ± 0.1[b] | 1.3 ± 0.2[a] |

Values are mean ± SEM expressed as the relative abundance (%) of bacterial taxa per total bacteria (16S rRNA gene copies of microbial group/total bacteria 16S rRNA gene copies). Mean values without a common letter are significantly different and denote a main effect of diet ($P<0.05$) when analyzed by repeated measures one-way ANOVA, where the between subjects factor was maternal diet and the within-subjects factor was timepoint (gestation days 1, 14 and 21 and lactation days 1 and 19). HFS, *n*= 11 except for *A. muciniphila* and *C. coccoides* where *n*=10; OFS, *n*=12; WM, *n*=9.

**Supplementary Table S3**. Significant associations with offspring body fat percent.

| **Variable** | **R*** |
| --- | --- |
| Maternal Percent Body Fat | 0.542† |
| Offspring *Methanobrevibacter* spp. abundance | -0.532† |
| Offspring Plasma Leptin | 0.395 |
| Offspring *A. muciniphila* abundance | 0.336 |
| Offspring *Bifidobacterium* spp. | -0.303 |
| Offspring *C. leptum* abundance | 0.274 |
| Offspring *Clostridium* cluster XI abundance | -0.244 |
| Offspring *Enterobacteriacea*e abundance | -0.226 |
| Offspring *Bacteroides/Prevotella* spp. abundance | -0.220† |
| Offspring *C. coccoides* abundance | -0.214 |

*All correlations $P<0.05$
† Pearson's correlation values

**Supplementary Table S4**. OPLS-DA model parameters

| Experimental Timepoint | Maternal Dietary Groups | Number of Samples | Cross-validation (k) | Number of Orthogonal Components | $R^2Y$* | $Q^2Y$** |
|---|---|---|---|---|---|---|
| **Baseline** | Obese vs. Lean | 53 | 7 | 1 | 0.677 | 0.518 |
| **Gestation** | HFS vs. OFS | 19 | 6 | 2 | 0.858 | 0.606 |
| **Gestation** | HFS vs. WM | 16 | 5 | 4 | 0.986 | 0.748 |
| **Gestation** | OFS vs. WM | 21 | 7 | 0 | 0.552 | 0.383 |
| **Lactation** | HFS vs. OFS | 19 | 6 | 0 | 0.800 | 0.710 |
| **Lactation** | HFS vs. WM | 18 | 6 | 0 | 0.845 | 0.785 |
| **Lactation** | OFS vs. WM | 17 | 5 | 0 | 0.689 | 0.469 |

*$R^2Y$ describes the amount of variation explained by the model
**$Q^2Y$ describes the predictability of the model

**Supplementary Table S5.** Experimental diet composition

| (g/kg) | HFS | HFS + 10% OFS (wt/wt) | AIN-93M | AIN-93G |
|---|---|---|---|---|
| **Cornstarch** | - | - | 465.7 | 397.5 |
| **Casein** | 200 | 180 | 140 | 200 |
| **Dyetrose** | - | - | 155 | 132 |
| **Sucrose** | 499.48 | 449.532 | 100 | 100 |
| **Soybean Oil** | 100 | 90 | 40 | 70 |
| **Lard** | 100 | 90 | - | - |
| **Alphacel** | 50 | 45 | 50 | 50 |
| **AIN-93M Mineral Mix** | 35 | 31.5 | 35 | 35 |
| **AIN-93-VX Vitamin Mix** | 10 | 9 | 10 | 10 |
| **L-cystine** | - | - | 1.8 | 3 |
| **DL-Methionine** | 3 | 2.7 | - | - |
| **Choline Bitartrate** | 2.5 | 2.25 | 2.5 | 2.5 |
| **OFS** | - | 100 | - | - |

HFS, High-fat/sucrose, 4.6 kcal/g; OFS, oligofructose, 4.28 kcal/g; AIN-93M, Lean control maintenance diet, 3.6 kcal/g; AIN-93G, Lean control diet for pregnancy and lactation, 3.76 kcal/g.
